# Automatic segmentation of large-scale CT image datasets for detailed body composition analysis

**DOI:** 10.1186/s12859-023-05462-2

**Published:** 2023-09-18

**Authors:** Nouman Ahmad, Robin Strand, Björn Sparresäter, Sambit Tarai, Elin Lundström, Göran Bergström, Håkan Ahlström, Joel Kullberg

**Affiliations:** 1 Department of Surgical Sciences, Radiology, Uppsala University, Uppsala, Sweden; 2 Department of Information Technology, Uppsala University, Uppsala, Sweden; 3 Department of Molecular and Clinical Medicine, Institute of Medicine, Sahlgrenska Academy, University of Gothenburg, Gothenburg, Sweden; 4 Department of Clinical Physiology, Sahlgrenska University Hospital, Region Västra Götaland, Gothenburg, Sweden; 5 Antaros Medical, Mölndal, Sweden

**Keywords:** Deep learning, Segmentation, Medical imaging, Computed tomography, Body composition

## Abstract

**Background:**

Body composition (BC) is an important factor in determining the risk of type 2-diabetes and cardiovascular disease. Computed tomography (CT) is a useful imaging technique for studying BC, however manual segmentation of CT images is time-consuming and subjective. The purpose of this study is to develop and evaluate fully automated segmentation techniques applicable to a 3-slice CT imaging protocol, consisting of single slices at the level of the liver, abdomen, and thigh, allowing detailed analysis of numerous tissues and organs.

**Methods:**

The study used more than 4000 CT subjects acquired from the large-scale SCAPIS and IGT cohort to train and evaluate four convolutional neural network based architectures: ResUNET, UNET++, Ghost-UNET, and the proposed Ghost-UNET++. The segmentation techniques were developed and evaluated for automated segmentation of the liver, spleen, skeletal muscle, bone marrow, cortical bone, and various adipose tissue depots, including visceral (VAT), intraperitoneal (IPAT), retroperitoneal (RPAT), subcutaneous (SAT), deep (DSAT), and superficial SAT (SSAT), as well as intermuscular adipose tissue (IMAT). The models were trained and validated for each target using tenfold cross-validation and test sets.

**Results:**

The Dice scores on cross validation in SCAPIS were: ResUNET 0.964 (0.909–0.996), UNET++ 0.981 (0.927–0.996), Ghost-UNET 0.961 (0.904–0.991), and Ghost-UNET++ 0.968 (0.910–0.994). All four models showed relatively strong results, however UNET++ had the best performance overall. Ghost-UNET++ performed competitively compared to UNET++ and showed a more computationally efficient approach.

**Conclusion:**

Fully automated segmentation techniques can be successfully applied to a 3-slice CT imaging protocol to analyze multiple tissues and organs related to BC. The overall best performance was achieved by UNET++, against which Ghost-UNET++ showed competitive results based on a more computationally efficient approach. The use of fully automated segmentation methods can reduce analysis time and provide objective results in large-scale studies of BC.

**Supplementary Information:**

The online version contains supplementary material available at 10.1186/s12859-023-05462-2.

## Introduction

Obesity is one of the key risk factors for the development of several cardiometabolic diseases, including type 2-diabetes (T2D), cardiovascular disease (CVD), non-alcoholic fatty liver disease and hypertension [1, 2]. Body composition (BC) analysis studies, the amounts and distribution of fatty and non-fatty tissues in different depots, including adipose tissue, muscle, liver and bone within the body. Accurate quantification of BC helps to understand cardiometabolic diseases and their prediction and prevention [3], with both total and regional adipose tissue being of importance. Adipose tissue compartment consists of visceral (VAT), subcutaneous (SAT), retroperitoneal (RPAT), intraperitoneal (IPAT), deep (DSAT), superficial SAT (SSAT), and intramuscular adipose tissue (IMAT). VAT is found in the intraabdominal region, surrounding intraabdominal tissues and organs. VAT can be separated into two sub-depots, RPAT and IPAT, the clinical significance of differentiating between IPAT and RPAT has been emphasized in [4, 5], with IPAT being linked to an increased risk of diabetes and both IPAT and RPAT having distinct associations with metabolic syndrome. Similarly, the SAT depot, which is located under the skin, can be separated into SSAT and DSAT. These depots have been found to contain different cell types and show differences in metabolic activity [3, 6–8].

For human BC analysis, several medical imaging techniques, such as magnetic resonance imaging (MRI) and computed tomography (CT), are often used [3]. These techniques are commonly adopted to quantify adipose tissue, muscle, and liver fat content in the body. The quantified adipose tissue measurements are often generated using manual or semi-automated image analysis techniques, which are usually time consuming and might give subjective results [9].

In the last decade, artificial intelligence (AI) has influenced many fields, with healthcare being one of the prime domains for which AI has shown remarkable performance. Various AI-based techniques have been developed to perform different tasks in the field of medicine. Due to automatic feature extraction and outcome prediction, deep learning has been widely adopted to solve various medical image analysis tasks [10]. Many deep learning-based techniques have been proposed for segmentation and BC analysis, including solutions for quantification of adipose tissue, muscle, and liver depots from CT images [11–14]. Typically, large amounts of data are required for the development of a deep learning model.

In this study, we used deep learning techniques to perform an advanced BC analysis on two large cohort studies; the Swedish CardioPulmonary bioImage Study (SCAPIS, n = 30,154) and the Impaired Glucose Tolerance Microbiota Study (IGT, n = 1965). SCAPIS [15] is a large-scale study that mainly focuses on analysing cardiovascular and pulmonary diseases, with CT angiography of the coronary arteries being the preferred technique. Similarly, the IGT [16] study aims to understand how the gut microbiota affects glucose dysregulation and cardiovascular disease development. Both studies include a 3-slice CT imaging protocol, which generates single axial slices at the level of the liver, abdomen, and thigh for quantification of BC. By restricting the image acquisition to three slices, the exposure of ionizing radiation to the subjects is reduced to only 0.245 mSv on average making the image acquisition protocol very attractive for large scale studies including healthy volunteers.

The aim of this study is to develop and evaluate fully automated segmentation techniques of various tissues and organs included in the SCAPIS and IGT cohort studies using the 3-slice (liver, abdomen, and thigh) CT imaging protocol. In order to achieve this goal, we propose four different deep learning architectures: ResUNET, UNET++, Ghost-UNET, and the novel Ghost-UNET++.

Our proposed method significantly reduces the need for manual annotation and enables efficient analysis of large-scale cohort studies of SCAPIS and IGT datasets, contributing to the field of medical image analysis by providing a robust and automated tool for accurate segmentation of complex anatomical structures in CT imaging. We conducted extensive experiments on a large, two cohort dataset of CT images and achieved remarkable performance in terms of segmentation accuracy.

Overall, the contributions of this study are twofold. First, we propose a novel deep learning architecture, Ghost-UNET++, and compare its performance with three existing architectures, ResUNET, UNET++, and Ghost-UNET, on the SCAPIS and IGT datasets. Second, we provide fully automated segmentation methods for a large number of targets of importance for body composition research that can be applied to large-scale studies of diverse patient populations, reducing the time and cost required for manual annotation.

## Material and methods

### Subjects

The study comprises two large-scale cohorts, SCAPIS and IGT. SCAPIS is a population-based CVD and chronic obstructive pulmonary disease (COPD) study (www.scapis.org) in which approximately 30,154 men and women aged between 50 and 64 years were randomly selected for a wide range of tests, including CT imaging for body composition analysis [15]. The image data were collected at six different university hospitals in Sweden between 2013 and 2018 (Uppsala, Stockholm, Malmö/Lund, Umeå, Linköping, and Gothenburg). The images used in the current study were chosen at random from the population recruited in Gothenburg. An initial random subset of this data was obtained to facilitate method development and evaluation. The complete multi-center SCAPIS data is being collected, compiled, and quality controlled and has yet not been shared with any research groups.

IGT [16] is a mirror cohort to SCAPIS, targeting subjects at risk of developing T2D and primarily aiming to understand how the gut microbiota affects glucose dysregulation and CVD development. The study includes about 1965 subjects with different forms of glucose dysregulation. The CT body composition imaging is identical to that of SCAPIS.

This present study was approved by the Swedish Ethical Review Authority (Dnr 2021-05856-01, Gothenburg, section 2, medicine), and all participants provided written, informed consent. The study was performed in accordance with relevant guidelines and regulations, including the Declaration of Helsinki.

### CT Protocol

**Fig. 1:**
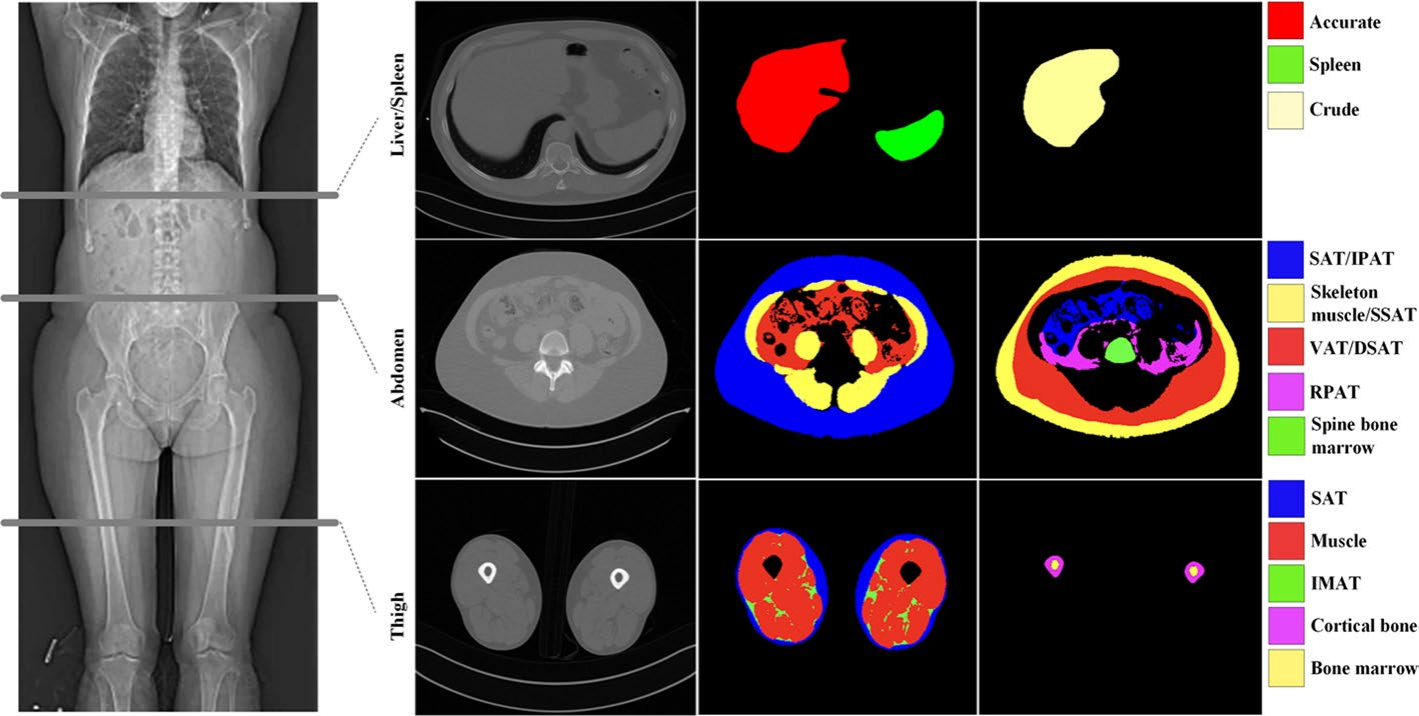
Illustration of the 3-slice CT images collected and the segmentation targets. The positioning of the three slices is shown on the CT scout to the left. Left column of axial images shows the tree slices in-plane (liver, abdomen, thigh). Middle and right column of axial images show resulting segmentation of liver, spleen and crude liver (top row), SAT/IPAT, skeleton, SSAT, VAT, DSAT, RPAT, spine bone marrow (middle row), SAT, muscle, IMAT, cortical bone, and bone marrow (bottom row)

Subjects in both SCAPIS and IGT were scanned with a non-contrast enhanced 3-slice CT imaging protocol for the liver, abdomen, and thighs, see (Fig. 1). The SCAPIS study and the CT protocol used have previously been thoroughly described [15]. Data acquisition was performed with the same CT scanner for all the subjects and procedures (Somatom Definition Flash with a Stellar detector, Siemens Healthcare, Forchheim, Germany) with slice thickness 5 mm, reconstruction kernel B31 medium smooth. For dose optimization, Care Dose 4D was employed.

### Reference segmentation

An overview of the reference segmentations used in the work is given in Table 1. The reference segmentations were created using different approaches and software.

**Table 1:** Overview of the images and reference segmentations used in the different experiments

Segmentation target	SCAPIS			IGT		
	Total	CV	Test	Total	CV	Test
**Liver slice**						
Liver accurate^IJ^	51	51	–	–	–	–
Liver crude^AM^	2681	2413	268	1951	1756	195
Spleen^DP^	51	51	–	–	–	–
**Abdomen slice**						
SAT^AM^	2677	2410	267	1951	1756	195
VAT^AM^	2677	2410	267	1951	1756	195
IPAT	1017	916	101	–	–	–
RPAT^IJ^	1017	916	101	–	–	–
DSAT^IJ^	529	477	52	–	–	–
SSAT	529	477	52	–	–	–
Spine bone marrow^DP^	208	188	20	–	–	–
Skeleton muscle^DP^	200	180	20	–	–	–
**Thigh slice**						
SAT^AM^	2682	2414	268	1951	1756	195
Muscle^AM^	2682	2414	268	1951	1756	195
IMAT^AM^	2682	2414	268	1951	1756	195
Bone marrow^a^	2683	–	–	1951	–	–
Cortical bone^a^	2683	–	–	1951	–	–

*Total *total number of images with references segmentation used, *CV* cross validation, *Test* test set used—the test split was only used when the total number samples were above 100

^AM^Reference segmentations generated at Antaros Medical, see “Methods” section

^IJ^Reference segmentations generated at Uppsala University using the software Image J, see “Methods” section

^DP^Reference segmentations generated at Uppsala University using the software Deep Paint, see “Methods” section

^a^Automatic segmentation pipeline developed with traditional image analysis techniques, without the use of deep learning, which is also evaluated separately

Most reference segmentations were generated based on manual corrections of results from an automated segmentation pipeline [3]. These were performed at Antaros Medical (AM) on the first batch of images from both SCAPIS and IGT for the purpose of quantification of the basic body composition parameters, i.e., liver fat, areas of VAT and SAT, as well as thigh muscle, SAT, and IMAT. An in-house constructed user interface was developed and used for efficient quality control and correction of all automated segmentations. The resulting segmentations were output as binary masks that were used in this study. The segmentation denoted “liver crude” was performed for the purpose of quantifying average liver attenuation. Therefore, a rapid delineation of the majority of the liver tissue was performed, not aiming for a detailed delineation of the entire liver area.

ImageJ (IJ) [17], was used for manual reference segmentation of the entire liver area and for creating the reference delineations of the outer contours of the DSAT and RPAT depots, hereafter denoted as the raw DSAT and the raw RPAT segmentations, respectively.

Deep Paint (DP) is a deep learning based 2D semi-automated segmentation tool, developed at Uppsala University and Antaros Medical, which can be used for efficient creation of reference segmentations. A built-in segmentation model (UNET) is used to generate a segmentation proposal. This proposal is then corrected by an expert and thereafter saved and used for re-training the segmentation model. Deep Paint was used to generate reference segmentations of the spleen, skeleton muscles, and spine bone marrow.

The generation of the reference segmentation masks for IPAT, RPAT, SSAT, DSAT, thigh bone marrow, and cortical bone is described below.

**Fig. 2:**
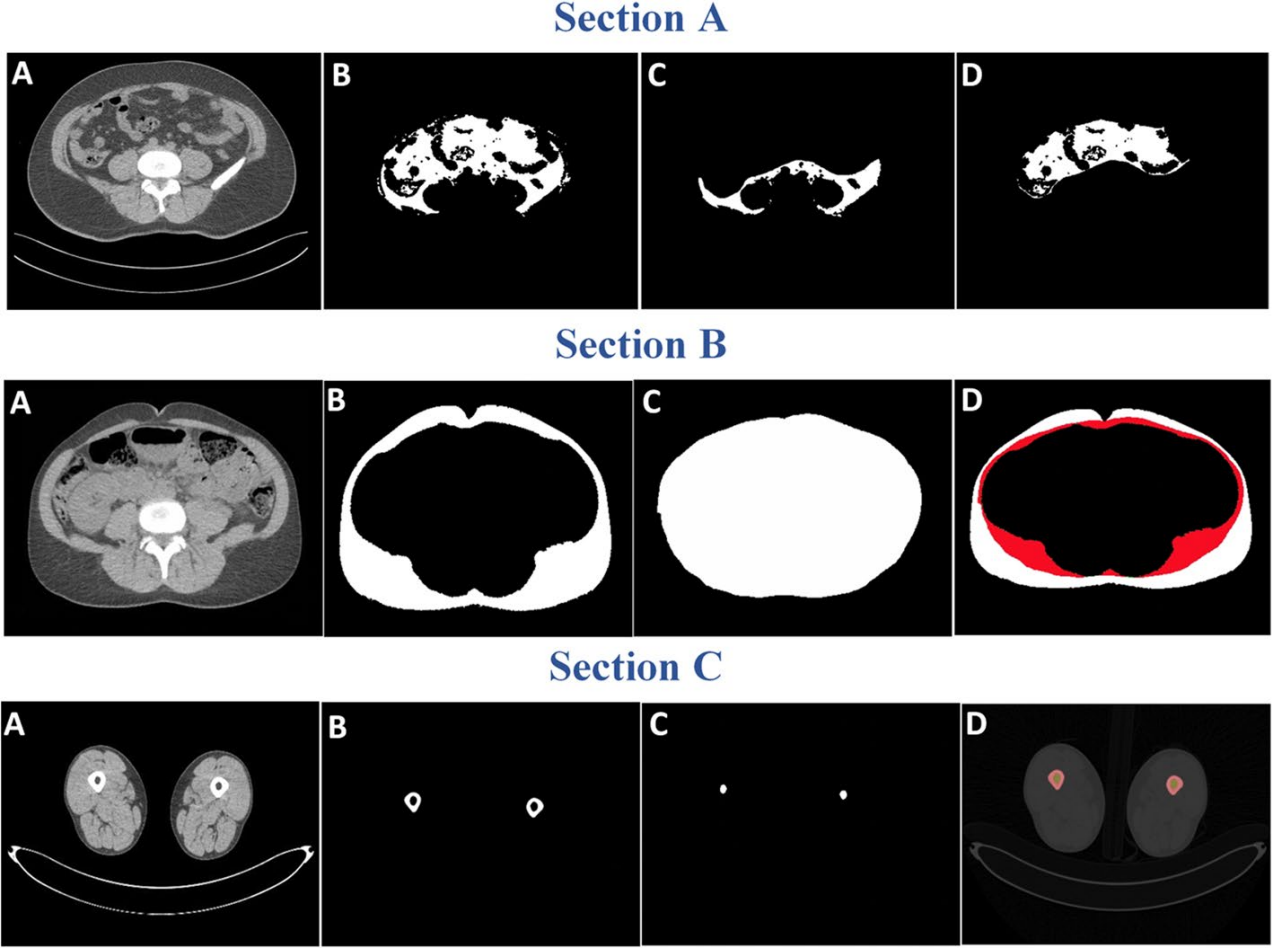
**Section A:** Illustration of IPAT mask generation, A (Abdomen CT slice), B (VAT) model output, C (RPAT) model output, D (IPAT). **Section B:** Illustration of DSAT and SSAT mask generation. A (Abdomen CT Slice), B (SAT) model output, C (Raw mask for DSAT), D (SSAT and DSAT). **Section C:** Illustration of segmentation of cortical bone and bone marrow. A (Thigh CT slice), B (Cortical bone), C (Bone marrow), D (segmented output overlayed on CT image)

For IPAT, RPAT, SSAT, and DSAT, the available raw RPAT and raw DSAT segmentations were combined, using basic mathematical operations, with segmentations of the entire VAT and SAT depots, respectively; see Fig. 2, Sections A and B.

The VAT and SAT segmentations were automatically generated by using a Ghost-UNET++ model trained on a large, non-overlapping dataset (n = 2677) of available VAT^AM^ and SAT^AM^ segmentations, respectively.

To segment the cortical bone and bone marrow in the thigh slices, an automatic segmentation pipeline was developed with traditional image analysis techniques without the use of deep learning. First the cortical bone region is segmented. This is done by applying a threshold on the voxel intensities > 400 Hounsfield Units (HU) [3] resulting in a binary image where cortical bone is segmented. Small segmented objects, from for example calcifications, were removed by filtering, and small holes inside the cortical region were filled.

The bone marrow segmentation was done by applying morphological operation on cortical segmented image to fill remaining two large holes containing bone marrow.

Finally, the cortical bone segmented image is subtracted from the morphological segmented image to obtain the target bone marrow segmentation. Representative example results are shown in Fig. 2 Section C.

The accuracy of the cortical bone and bone marrow segmentations was assessed through a visual examination of 210 randomly chosen thigh slices from the SCAPIS study and 185 thigh slices from the IGT study. Any discrepancies in the segmentation, such as errors, anatomical deviations, or outliers, were recorded.

### Data pre-processing

All CT image data underwent three pre-processing steps prior to being used to train and evaluate a deep learning model. HU windowing was used to limit the voxel intensity range for each slice of liver, abdomen, and thigh. Different fix ranges were tested and evaluated for the different segmentation targets. The final HU ranges for image slices used were liver [− 25, 125], abdomen [− 219, 190], thigh [− 198, 189], skeletal muscle and spine bone marrow segmentation were [− 181, 216]. An adaptive median filtering algorithm [18] was applied to reduce noise without significantly blurring important structures. Image intensities were normalized image-wise using z-score normalization [19].

### Proposed deep learning models

To perform segmentation tasks, several deep learning models based on convolutional neural network (CNN) have been proposed. The majority of these techniques were based on pretrained architectures that required a specific weight file. In this study, we proposed a novel deep learning architecture, Ghost-UNET++, based on the nested UNET model by substituting convolutional layers with the so-called Ghost module, with the aim of getting more feature maps with cheaper operations. We also compared the proposed network with three other deep learning architectures: ResUNET, UNET++, and Ghost-UNET.

The ResUNET network is a widely used network consisting of a convolution layer followed by Relu, max pooling, and batch normalization, along with a skip connection (see detailed description in section ResUNET).

The UNET++ architecture is made up of nested architectures with redesigned skip connections to reduce the semantic gap between encoder and decoder feature maps. UNET++ consists of convolution layers followed by Relu and batch normalization. Each convolutional layer is connected with other layers in the nested block (see detailed description in section UNET++).

In 2021, authors proposed a Ghost-UNET [20]**,** based on an asymmetry encoder-decoder architecture with the combination of UNET and Ghost-modules. This study presents the Ghost-UNET++ network, which combines UNET++ with the recently proposed Ghost module. In this approach, the convolutional blocks of the UNET++ architecture are replaced with Ghost-modules.

#### ResUNET

UNET is a deep learning-based fully convolution neural network for fast and accurate medical image segmentation [21]. To enhance the performance of the UNET architecture, a ResUNET model [22] was proposed, in which the traditional convolutional blocks are substituted with residual blocks. The residual block has identity mapping to add the output feature map of the previous layer to the next layer.

1 $${\varvec{z}} = {\mathcal{F}}\left( {x,\left\{ {W_{a} } \right\}} \right) + W_{s} x.$$

**Fig. 3:**
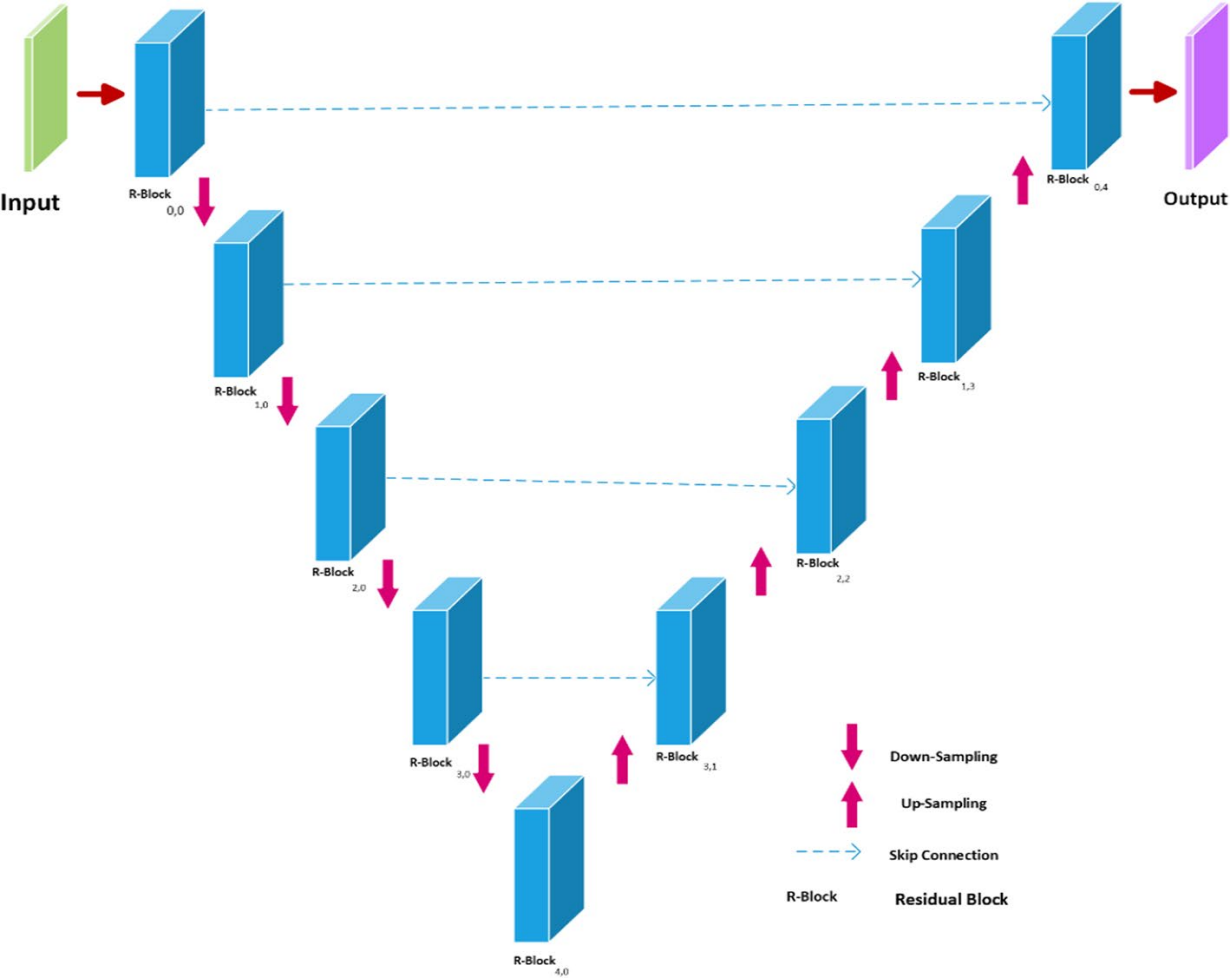
ResUNET architecture

Equation 1, shows the building block of ResUNET, where ${\mathcal{F}}\left( x \right)$ feature map and x identity mapping are multiplied by a linear project W to expand the channels of shortcut to match the residual. The ResUNET architecture used in this study is illustrated in (Fig. 3).

#### UNET++

The authors [23] proposed a UNET++ architecture to ameliorate the UNET model. In the UNET++ network, a series of nested blocks are linked together to reduce the semantic gap between the contraction and expansion paths.

**Fig. 4:**
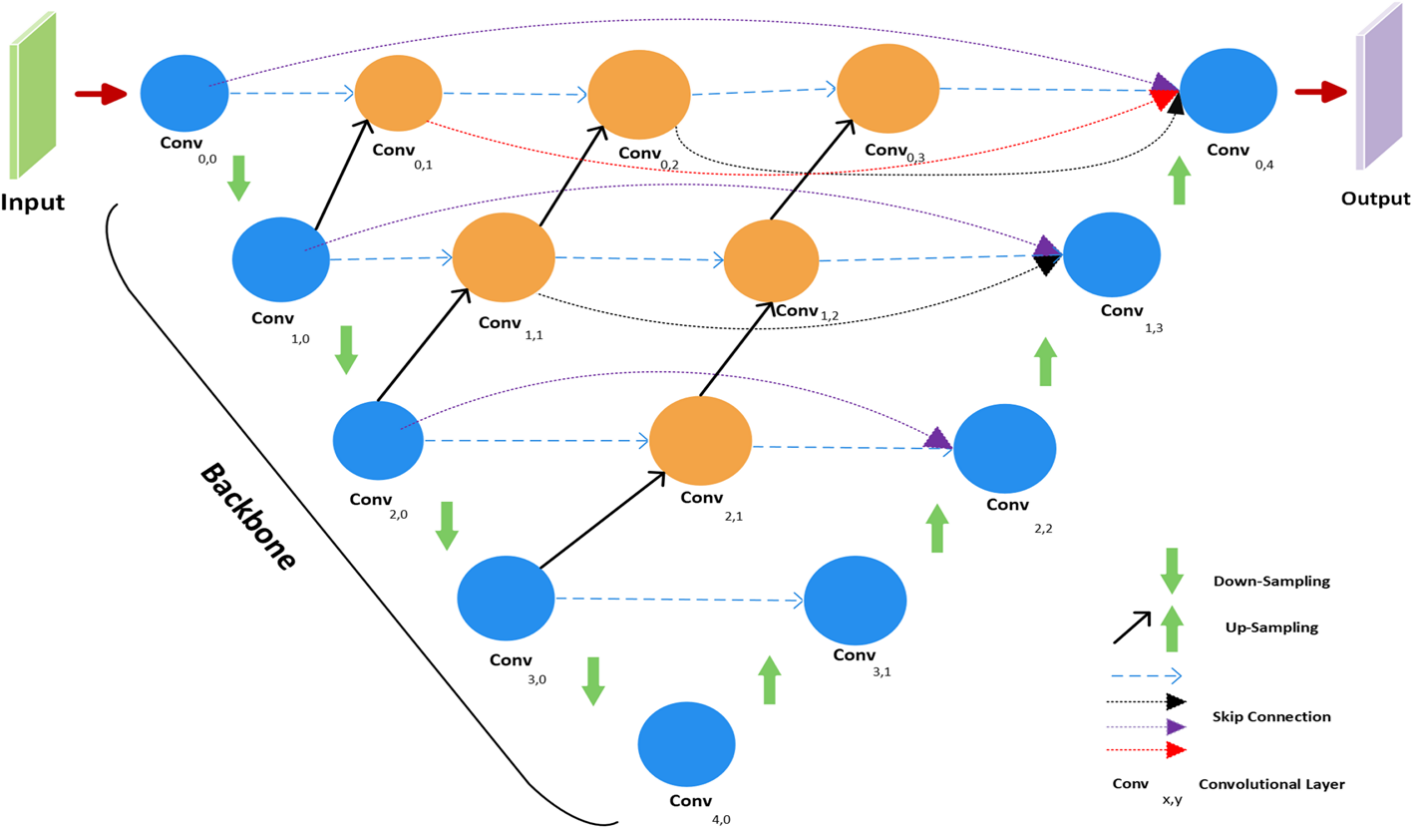
UNET++ architecture

The entire network consists of nested blocks that are connected in a series, with each block of the network consisting of two convolution layers followed by batch normalization and Relu, with the purpose of generalizing model performance. The max pooling and upsampling layers are adopted in a way to extract prime features and remap features to generate segmentation maps. Finally, a convolution layer followed by a sigmoid activation map is added to predict the final outcomes. The UNET++ architecture used in this work is illustrated in (Fig. 4).

#### Ghost-UNET++

The Ghost-Net architecture is described as extracting more intrinsic features from cheap operations. The aim of the Ghost-Net model is to design an efficient convolution neural network by reducing the redundancy in feature maps. However, simple convolutions are computationally expensive for generating feature maps. The Ghost-Net module uses cheaper operations to generate the feature maps. In the Ghost-Net architecture, each layer is made up of a bottleneck, which is made by stacking two Ghost modules [24].

The Ghost module is a feature in the Ghost-Net architecture that helps improve the network’s performance without adding too many parameters. It works by using ghost features, which are low-resolution versions of the input feature maps. Equation 2, the Ghost module:

2 $$y = \gamma \cdot\,x_{high} + \beta \,\cdot\,GhostConv\left( {x_{low} ,W} \right)$$

where $x_{high}$ is the high-resolution feature map, $x_{low}$ is the low-resolution ghost feature map, *W* is the weight tensor for the ghost convolution layer, *γ* and *β* are learnable scale and shift parameters, and $GhostConv$ is the ghost convolution operation.

The ghost convolution operation is defined as follows Eq. 3:

3 $$GhostConv\left( {x_{low} ,W} \right)_{i,j,k} = \mathop \sum \limits_{l = 1}^{{n_{ghost} }} W_{i,j,k,l} .x_{{low_{i \times s,j \times s,l} }}$$

where $n_{ghost}$ is the number of ghost channels, *s* is the stride, and $x_{{low_{x \times s,j \times s,l} }}$ is the value at position $i \times s,j \times s,l$ in the low-resolution feature map.

In this study, we designed a novel Ghost-UNET++ architecture by substituting convolution layers with Ghost bottleneck layers in the UNET++ model. The proposed network consists of 15 bottleneck layers connected in a series of nested architectures to build a Ghost-UNET++ model. The aim of the network is to reduce semantic gaps and redundancy in feature maps, hence improving network performance based on the UNET++ method. The proposed architecture is based on contraction and expansion paths to perform segmentation tasks. Each block in a path consists of a Ghost bottleneck layer stacked with two Ghost-Net models.

Here’s the UNET++ model with Ghost modules expressed in a mathematical form in Eq. 4:

4 $${\varvec{y}} = {\mathcal{F}}\left( {{\mathcal{X}};\Theta } \right),$$

where ${\mathcal{X}}$ is the input tensor, $y$ is the output tensor, and ${\Theta }$ represents the set of learnable parameters of the model.

Each level $i$ of the UNET++ model with Ghost modules is defined by the following functions:

5 $$x_{i} = GhostDown\left( {x_{i - 1} } \right), d_{i} = Pool\left( {x_{i} } \right), y_{i} = GhostUp\left( {d_{i,} u_{i - 1} } \right), u_{i} = Upconv\left( {y_{i} } \right),$$

In Eq. 5, where $x_{i - 1}$ is the input feature map from the previous level, $d_{i}$ is the down-sampled feature map, $u_{i - 1}$ is the up-sampled feature map from the corresponding level of the down-sampling path, and $y_{i}$ is the output feature map of the current level.

The $GhostDown,GhostUp,Pool, and Upconv$ functions represent the Ghost module, pooling operation, and up-convolutional operation, respectively.

**Fig. 5:**
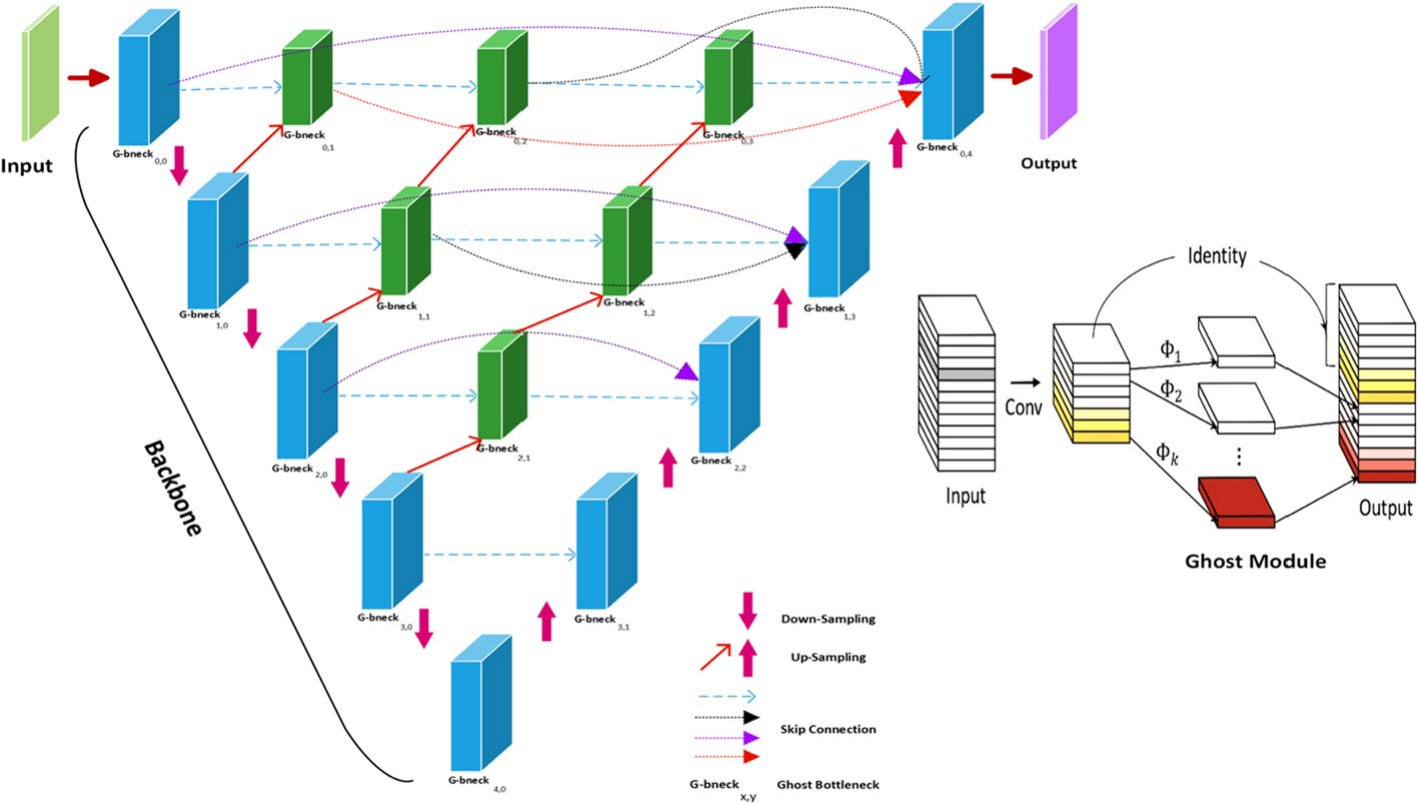
Ghost-UNET++ architecture

The final output of the UNET++ model with Ghost modules is given by $y = y_{n}$, where *n* is the number of levels in the model. The entire network is connected in a series of nested layers as illustrated in (Fig. 5).

#### Experimental settings

To allow fair comparison of the networks’ performance, all four networks were configured uniformly. The following settings were used in the conducted experiments:

1. All the experiments were performed on a Linux platform and a Nvidia GeForce RTX 2080Ti, 11 Gigabyte of GPU memory.
2. The Pytorch framework was used for each network implementation and experiment.
3. The batch size was set to 2 with an input dimension of 512 × 512 × 1.
4. The Adam [25] optimizer was used with the learning rate set to 0.0001, learning weight initialized with default settings.
5. All networks were trained for a maximum of 100 epochs. Early stopping was used to reduce overfitting.
6. A tenfold cross validation was used to train and evaluate the models.
7. All networks were trained from scratch, without the use of pre-training.
8. 10% of the images were set aside for testing. This was done for segmentation targets where the number of samples available was greater than 100. The best performing model from the tenfold cross validation was further tested.
9. Dice loss was used with a smoothing factor added to numerator and denominator. This is needed to handle for example non-overlapping predicted and ground truth segmentations and ensures numerical stability and prevents for example division by zero. The Dice loss between ground truth and prediction is presented below.
  6 $$DiceLoss\left( {A,B} \right) = 1 - \frac{{2X\left| {A \cap B} \right| + smooth}}{\left| A \right| + \left| B \right| + smooth}$$

Equation 6, where A is the set of input voxels in the target reference (ground truth) and B is the set of voxels in the prediction segmentation and smoothing factor = 1.0.

To ensure that each target is captured optimally, we designed individual models for each one. The models were trained to recognize specific characteristics of each target by learning from a large number of input images. To enable a better capture of the relevant features, we designed the final convolution layer of the networks to produce an output tensor with a dimension of 512 × 512 × 1. We followed this by applying a sigmoid activation function to the output tensor. This function transformed the output into a probability distribution, ranging from 0 to 1. By doing this, we were able to interpret the model's output as the probability of the target being present in the input image. To avoid overfitting and enhance the network's performance and stability, a batch normalization layer was added [26] in each layer of the network before applying the nonlinear transformation (ReLU). Furthermore, zero padding was applied throughout the network to ensure that the output feature map generate same dimension as the input dimension.

## Results

**Fig. 6:**
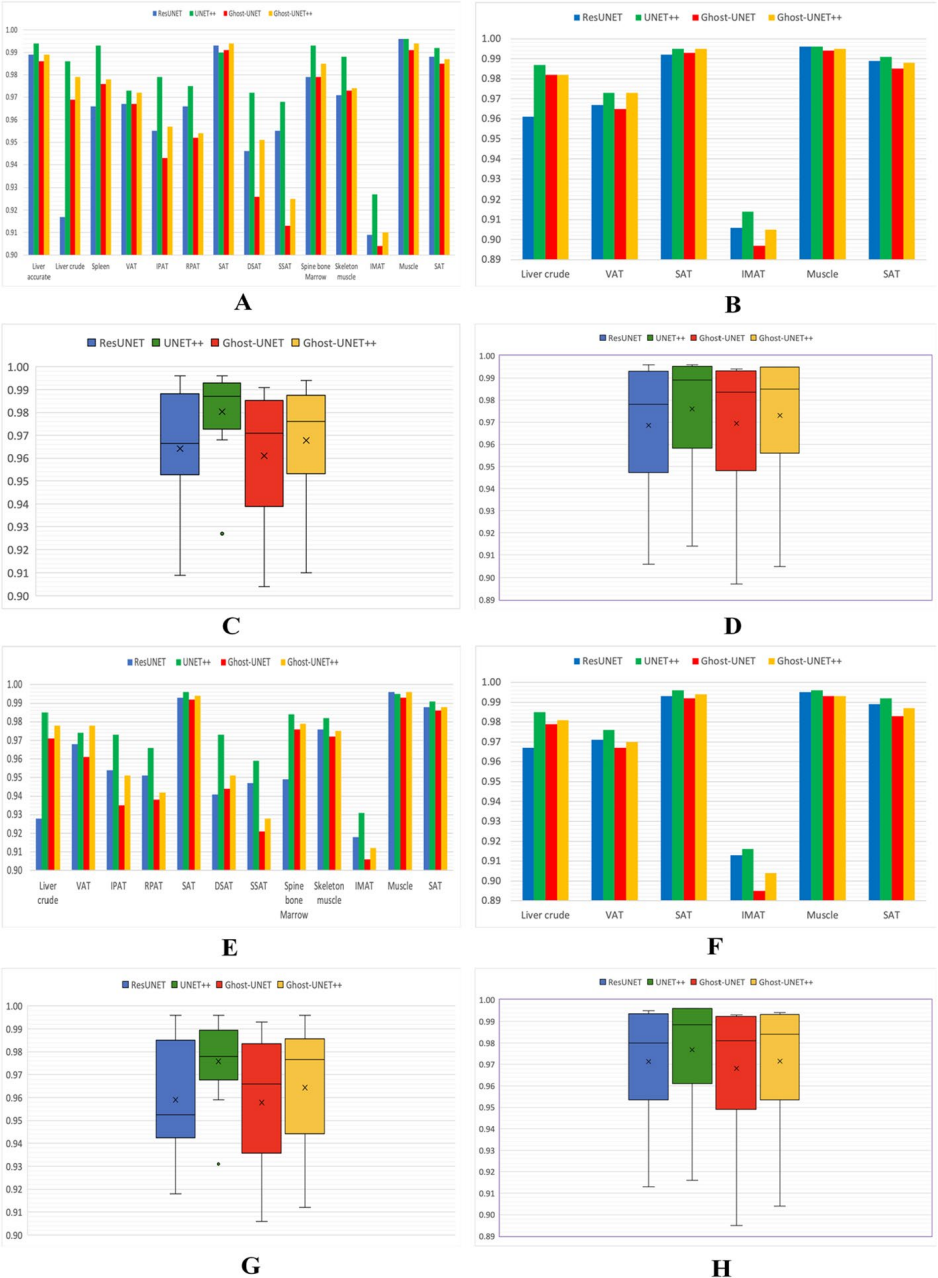
Illustration of Dice scores and average Dice scores for SCAPIS (**A**, **C**, **E**, **G**) and IGT (**B**, **D**, **F**, **H**). **A**, **B**, **C**, **D** plots represent the cross validation results and **E**, **F**, **G**, **H** plots represents the test set results

The experimental outcomes of the above-mentioned CNN models are presented in Table 2 and Fig. 6. Overall, the models exhibited good agreement with the ground truth for both cross validation and test sets. In cross validation outcomes, the Dice scores of our proposed Ghost-UNET++ network for spleen, liver, abdomen, and thigh slices were found to be between 0.910 and 0.994, respectively.

**Table 2:** Mean segmentation Dice score for the different targets and evaluations in both SCAPIS and IGT Datasets

Segmentation target	Experimental results															
	SCAPIS								IGT							
	ResUNET		UNET++		Ghost-UNET		Ghost-UNET++		ResUNET		UNET++		Ghost-UNET		Ghost-UNET++	
	CV	Test	CV	Test	CV	Test	CV	Test	CV	Test	CV	Test	CV	Test	CV	Test
**Liver slice**																
Liver accurate	0.989	–	**0.994**	–	0.986	–	0.989	–	–	–	–	–	–	–	–	–
Liver crude	0.917	0.928	**0.986**	**0.985**	0.969	0.971	0.979	0.978	0.961	0.967	**0.987**	**0.985**	0.982	0.979	0.982	0.981
Spleen	0.966	–	**0.993**	–	0.976	–	0.978	–	–	–	–	–	–	–	–	–
**Abdomen slice**																
VAT	0.967	0.968	**0.973**	0.974	0.967	0.961	0.972	**0.978**	0.967	0.971	**0.973**	**0.976**	0.965	0.967	**0.973**	0.970
IPAT	0.955	0.954	**0.979**	**0.973**	0.943	0.935	0.957	0.951	–	–	–	–	–	–	–	–
RPAT	0.966	0.951	**0.975**	**0.966**	0.952	0.938	0.954	0.942	–	–	–	–	–	–	–	–
SAT	0.993	0.993	0.990	**0.996**	0.991	0.992	**0.994**	0.994	0.992	0.993	**0.995**	**0.996**	0.993	0.992	**0.995**	0.994
DSAT	0.946	0.941	**0.972**	**0.973**	0.926	0.944	0.951	0.951	–	–	–	–	–	–	–	–
SSAT	0.955	0.947	**0.968**	**0.959**	0.913	0.921	0.925	0.928	–	–	–	–	–	–	–	–
Spine bone marrow	0.979	0.949	**0.993**	**0.984**	0.979	0.976	0.985	0.979	–	–	–	–	–	–	–	–
Skeleton muscle	0.971	0.976	**0.988**	**0.982**	0.973	0.972	0.974	0.975	–	–	–	–	–	–	–	–
**Thigh slice**																
IMAT	0.909	0.918	**0.927**	**0.931**	0.904	0.906	0.910	0.912	0.906	0.913	**0.914**	**0.916**	0.897	0.895	0.905	0.904
Muscle	**0.996**	**0.996**	**0.996**	0.995	0.991	0.993	0.994	**0.996**	**0.996**	0.995	**0.996**	**0.996**	0.994	0.993	0.995	0.993
SAT	0.988	0.988	**0.992**	**0.991**	0.985	0.986	0.987	0.988	0.989	0.989	**0.991**	**0.992**	0.985	0.983	0.988	0.987
Average dice score	0.964 ± 0.025	0.959 ± 0.024	**0.981 ± 0.017**	**0.976 ± 0.017**	0.961 ± 0.028	0.958 ± 0.027	0.968 ± 0.025	0.964 ± 0.026	0.968 ± 0.031	0.972 ± 0.028	**0.976 ± 0.028**	**0.977 ± 0.028**	0.969 ± 0.034	0.968 ± 0.034	0.973 ± 0.032	0.972 ± 0.032

Mean dice score of the respective targets and experiments is presented. Average dice scores are in (mean ± standard deviation) and boldface represents the best scores. The models were trained and validated individually for each reference segmentation target

*CV *cross validation

In general, results achieved by four models during cross validation on the SCAPIS cohort had mean Dice scores of 0.964 (min 0.909 and max 0.996) for ResUNET, 0.981 (0.927–0.996) for UNET++, 0.961 (0.904–0.991) for Ghost-UNET, and 0.968 (0.910–0.994) for Ghost-UNET++. Similarly, for the IGT cohort, the mean Dice scores for the ResUNET model were 0.968 (0.906–0.996), UNET++ 0.976 (0.914–0.996), Ghost-UNET 0.969 (0.897–0.994), and Ghost-UNET++ 0.973 (0.905–0.995). These findings indicate that UNET++ obtained the highest nominal Dice score in 26 out of 28 comparisons for SCAPIS and in 10 out of 12 comparisons for IGT.

On a given set of test data, the ResUNET achieved a maximum Dice score of 0.993 for abdominal SAT on SCAPIS and 0.996 for thigh muscle on IGT data. The UNET++ model, the maximum Dice score for abdominal SAT was 0.996 for SCAPIS and thigh muscle was 0.996 for IGT test data. Similarly, the Ghost-UNET model, the maximum Dice score for thigh muscle was 0.993 for both SCAPIS and IGT test data. The proposed Ghost-UNET++ achieved a maximum Dice score of 0.996 for SCAPIS thigh muscle on test data and 0.995 for IGT abdominal SAT and thigh muscle on the cross validation. However, for the thigh IMAT including both cross validation and test data, the networks performance was found to be comparatively lower, ranging between 0.895 and 0.931.

Based on our findings, the experimental outcomes indicate that UNET++ and Ghost-UNET++ outperformed ResUNET and Ghost-UNET in terms of the average Dice score. UNET++ demonstrated slightly better performance for all segmentation tasks, however the proposed Ghost-UNET++ model exhibited competitive performance with fewer trainable parameters. Table 3 presents a comparison of ResUNET, UNET++, and Ghost-UNET++ in terms of the trainable parameters and memory required by each network. The results of the comparison of models in terms of mean Dice score and average Dice score are shown in Fig. 6 for both the SCAPIS and IGT cohorts.

**Table 3:** Parameters and model size comparison of models

Network	Trainable parameters (M)	Model size (MB)
ResUNET	0.81	3.3
UNET++	2.29	9.3
Ghost-UNET++	0.35	1.7

**Fig. 7:**
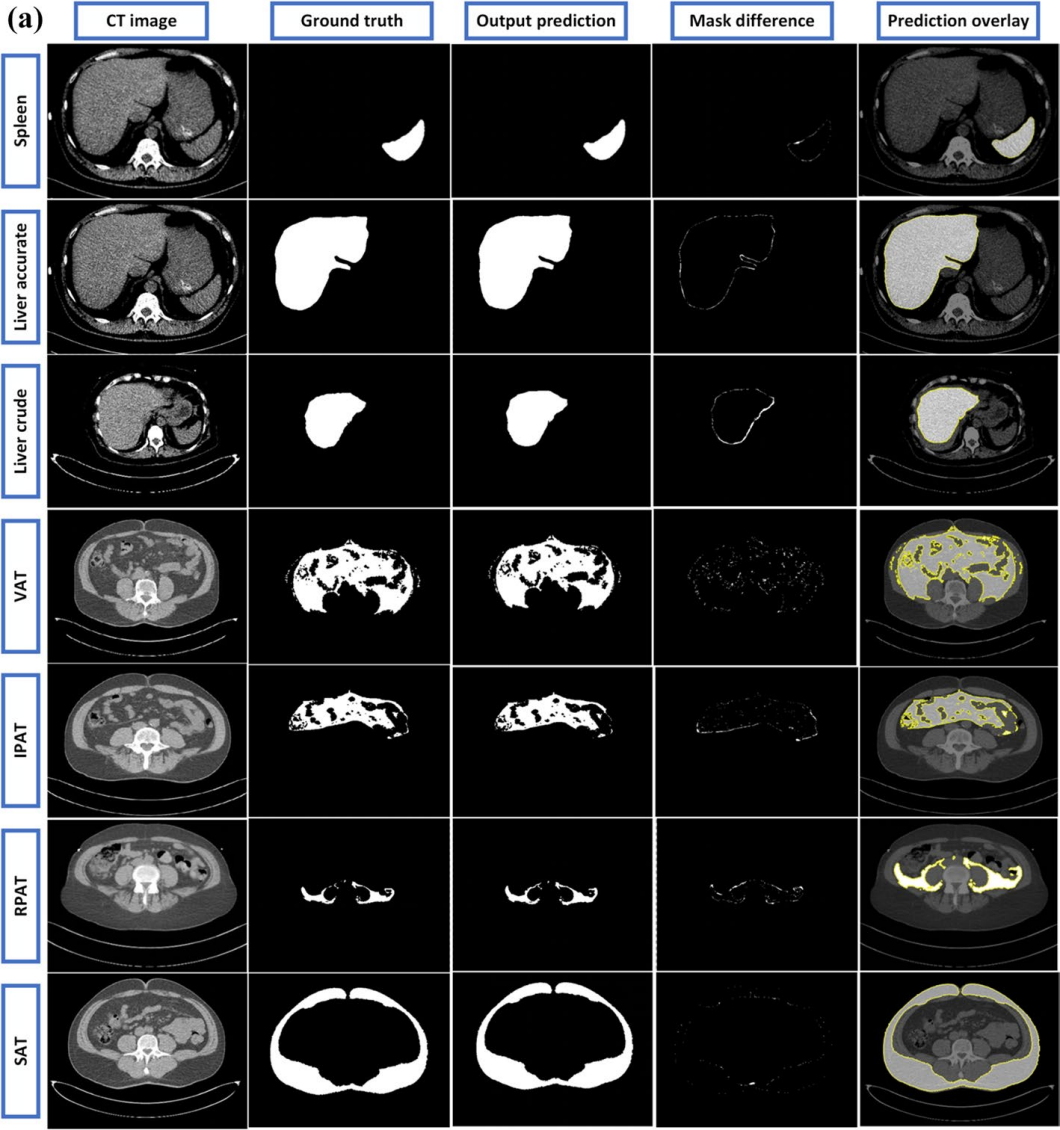

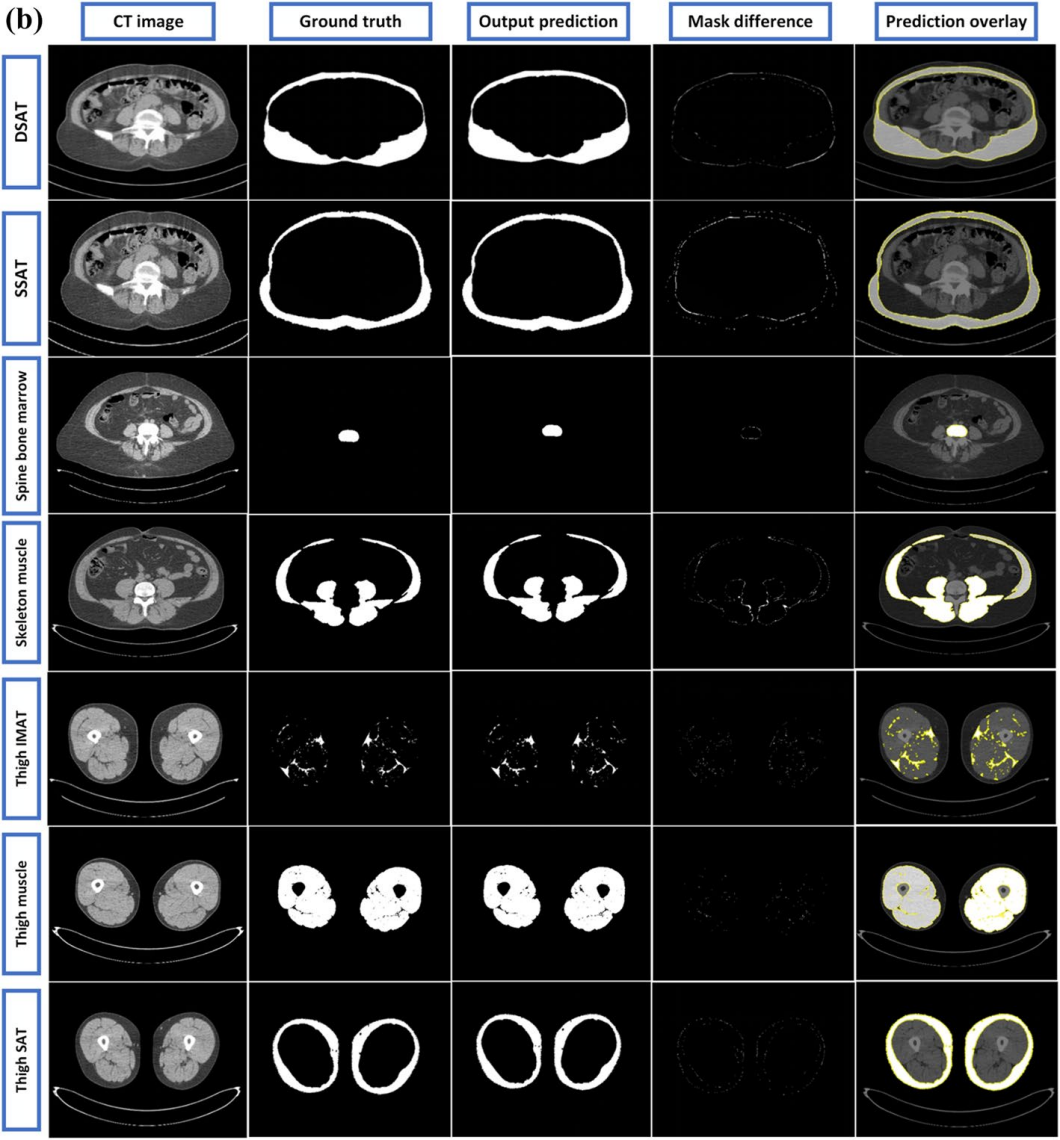
**a** Illustration of UNET++ model predictions and comparison to reference segmentations for randomly selected CT image examples, from top to bottom (Spleen to SAT) images: from left to right, CT image, ground truth, models predicted output, mask difference between ground truth and prediction, predicted mask overlayed on the original CT image, highlighted segmented region (contour) with mark boundaries. **b** Illustration of UNET++ model predictions and comparison to reference segmentations for randomly selected CT image examples, from top to bottom (DSAT to Thigh SAT) images: from left to right, CT image, ground truth, models output prediction, mask difference between ground truth and prediction, predicted mask overlayed on the original CT image, highlighted segmented region (contour) with mark boundaries

The predicted outcomes of the UNET++ network is illustrated in (Fig. 7a, b). The results from the ResUNET, Ghost-UNET, and Ghost-UNET++ networks are in addition illustrated in Additional file 1 (Fig. S2(a), S2(b)), (Fig. S3(a), S3(b)), and (Fig. S4(a), S4(b)). The figures demonstrate that the network's predictions are well generalized and can accurately predict the organs and fat regions for each segmentation task. The results further indicate that the models have learned important anatomical features to enable accurate predictions for highly ambiguous regions. In summary, the network's predictions are highly accurate and demonstrate a robust ability to generalize the results to a range of anatomical features.

During the visual assessment of the cortical bone and bone marrow segmentations obtained from the SCAPIS and IGT studies, only one participant from each study was identified to have quality issues. The cause of these issues was attributed to anatomical anomalies in the images, specifically, the absence of cortical bone structure in one image, and the presence of a probable metal implant in the other. Additional file 1, specifically (Fig. S1), depict the anomalous images.

## Discussion

Deep learning techniques for segmentation of numerous tissues and organs have been developed and evaluated, allowing for detailed analysis of body composition from a 3-slice CT imaging protocol. These techniques can reduce analysis time and give objective results, with significant benefits, especially in large-scale studies. CT-slice images from more than 4000 subjects at the level of the liver, abdomen, and thigh were from the SCAPIS and IGT cohort studies were utilized. The study comprised four fully convolutional architectures; ResUNET, UNET++, Ghost-UNET, and the proposed Ghost-UNET++, which were trained, validated, and compared using similar configurations.

Based on our experiments, we found that all four fully convolutional architectures—ResUNET, UNET++, Ghost-UNET, and Ghost-UNET++—had good overall performance for segmentation of multiple tissues and organs. The Dice scores achieved by the networks ranged from 0.895 to 0.996, with the thigh muscle segmentation obtaining the highest score and the IMAT segmentation obtaining the lowest score. This is likely because IMAT has a relatively small target area and high inter-subject variability.

UNET++ architecture outperformed ResUNET, Ghost-UNET, and Ghost-UNET++ in terms of overall segmentation performance. Specifically, it achieved the highest mean Dice scores in 26 out of 28 comparisons in the SCAPIS cohort and in 10 out of 12 comparisons in the IGT cohort. ResUNET had a comparatively lower segmentation Dice score for crude liver segmentation in the SCAPIS cohort, which may be because the model was unable to generalize the complex nature of the data.

The experimental results showed that all four fully convolutional architectures had remarkable performance for segmentation without requiring any further correction. However, the UNET++ model had slightly better overall performance compared to the other three models, as demonstrated in (Fig. 7a, b).

Although the proposed Ghost-UNET++ architecture had good performance with a small number of trainable network parameters and was capable of generating more feature maps with cheaper operations, that conclude the network was computationally inexpensive [24]. Specifically, it achieved high segmentation accuracy with a lower computational cost compared to the other models. These results suggest that the Ghost-UNET++ architecture may be a useful option for scenarios where computational resources are limited. Notably, the performance difference between the Ghost-UNET++ and UNET++ models was very small. In cross validation, the Ghost-UNET++ model achieved on average only 0.013 and 0.003 lower Dice scores for SCAPIS and IGT, respectively, which in some settings might be acceptable. We also compared the segmentation performance of Ghost-UNET++ with Ghost-UNET, on both SCAPIS and IGT data. Ghost-UNET++ showed higher mean Dice scores for all targets on both cross validation and test set.

The experiments for each network were conducted on the same configuration settings, where the number of kernels was set to [16,32,64,128,256] from top to bottom layers. The trainable parameters and memory utilization of each network under these settings were significantly different, as shown in Table 3. Our experiments revealed that the Ghost-UNET++ achieved a relatively high Dice score despite having fewer trainable parameters, indicating that it is computationally cost-effective and memory-efficient. In spite of network comparison, these four architectures also allowed the separation of VAT into IPAT and RPAT, as well as SAT into DSAT and SSAT, respectively. These four fat depots are relevant to quantifying as they manifest distinct biological and morphological characteristics, respectively [3, 14].

This study's findings indicate that development and evaluation of fully automated segmentation techniques applicable to a 3-slice CT imaging protocol demonstrates the potential clinical effectiveness of reducing analysis time and providing objective results in large-scale studies of body composition, potentially contributing to a better understanding of the relationship between body composition and disease risk.

We conducted a comparison of our findings with prior literature by identifying 17 studies with comparable imaging protocols and segmentation targets (listed in Table 4). These studies encompassed liver, abdomen, and thigh CT and MR imaging data, as well as investigations that assessed different segmentation targets, typically with fewer measurements than our study.

**Table 4:** Literature review references for included segmentation targets

Target	Architecture (performance)
Liver	2D EfficientNet based UNET [27] (0.960); PADLL [28] (0.965); *UNET++ (0.994)
Spleen	2D EfficientNet based UNET [27] (0.950); *UNET++ (0.993)
VAT	UNET [11] (0.940); FCN-based Segmentation [12] (0.970); UNET (pretrain VGG-16 encoder) [14] (0.960); 3D(RGA-UNET) and Standard 3D UNET [29] (0.90) Automatic segmentation method [30] (0.955); CNN (encoder and decoder) [31] (0.970); FatSegNet [32] (0.990); UNET [33] (0.968); Fast graph-based algorithm [34] (0.996); UNET [35] (0.997); UNET [36] (0.9746); Fully automatic segmentation algorithm [37] (0.920); UNET [38] (0.970); *UNET++ (0.973)
SAT	UNET [11] (0.940); FCN-based Segmentation [12] (0.970); Automatic segmentation method [30] (0.972); CNN (encoder and decoder) [31] (0.980); FatSegNet [32] (0.990); UNET [33] (0.968); Fast graph-based algorithm [34] (0.996); UNET [35] (0.998); UNET [36] (0.943); UNET [38] (0.960); UNET [39] (0.970); UNET [40] (0.976); *Ghost-UNET++ (0.994)
DSAT/SSAT	UNET (pretrain VGG-16 encoder) [14] (0.909/0.960); 3D(RGA-UNET) and Standard 3D UNET [29] (0.880/0.920); Fully automatic segmentation algorithm [37] (0.820/0.880); *UNET++ (0.972/0.968)
Spine bone marrow	UNET [39] (0.920); *UNET++ (0.993)
Skeleton muscle	FCN-based segmentation [12] (0.970); automatic segmentation method [30] (0.952); UNET [39] (0.950); *UNET++ (0.988)
IMAT	CNN (encoder and decoder) [31] (0.830); UNET [39] (0.910); *UNET++ (0.927)

In certain articles, multiple scores were obtained for each segmentation target. However, only the highest score value for each target reported. The performance is given in terms of Dice/Jaccard score. The Dice score from the best performing model from this work is also included for simplified comparison

*Represents the cross validation performance on SCAPIS achieved in the present study

In our analysis, we found that the Dice scores for liver (mean Dice score 0.963/max 0.965, from n = 2 papers), spleen (Dice score 0.95, from n = 1 paper), VAT(mean Dice score 0.963/max 0.997, from n = 13 papers), SAT(mean Dice score 0.972/max 0.998, from n = 12 papers), DSAT (mean Dice score 0.869/max 0.909, from n = 3 papers), SSAT(mean Dice score 0.920/max 0.960, from n = 3 papers), spine bone marrow(Dice score 0.920, from n = 1 papers), skeleton muscle(mean Dice score 0.957/max 0.970, from n = 3 papers), and thigh IMAT (mean Dice score 0.870/max 0.910, from n = 2 papers), respectively. This comparison shows that we present the top scoring performance in mean Dice scores for all targets but two (VAT and SAT) of the target measures. These targets also have the most previous studies found. The scores presented in this work are however above the means of the reported scores for both these targets.

Prior to the application of deep learning algorithms in our study, we conducted preliminary experiments to evaluate the effectiveness of various pre-processing techniques. These techniques included Gaussian filters, median filters, and data augmentation methods such as rotation, scaling, translation, flip, and volumetric deformations. However, we found that none of these methods resulted in improved outcomes compared to the pre-processing method we ultimately adopted, which involved the use of an adaptive median filter and intensity scaling.

Cortical bone and bone marrow segmentation in thigh could be achieved with simple hand-crafted methods including intensity thresholding and morphological operations. These methods were able to accurately segment the region, except for a few samples that required manual correction. This finding suggests that simple methods can be effective in cases where deep learning algorithms may not be necessary or practical. These results may have implications for the development of simpler and more efficient segmentation techniques that are accessible and widely applicable.

### Limitations

In spite of the aforementioned, there are a set of limitations to our study. Firstly, the 3-slice CT images used in our study was collected in Gothenburg, whereas the full SCAPIS cohort dataset was collected in six university hospitals throughout Sweden. Although the imaging was performed using standardized equipment and protocols. We therefore expect similar performance in SCAPIS, CT images from other centres.

Secondly, in our imaging protocol, the fascia of Scarpa, which separates DSAT and SSAT depots, is not visible in all abdomen CT scans. Consequently, reference segmentations were performed only in scans where the entire fascia was identifiable and delineated, which amounted to 61.29% of the total subjects. The performance of the segmentation algorithm in scans where the fascia is not visible is therefore not known and cannot be evaluated using the image data collected. Future applications will require an initial classification step where CT images with visible fascia are first identified before applying the segmentation tasks.

Thirdly, for the segmentations of IPAT, RPAT, SSAT and DSAT, Ghost-UNET++ was used for creation of the VAT and SAT masks needed for the creation of the reference masks. This has likely benefitted the evaluations of Ghost-UNET++ over other networks for these four target measurements.

Lastly, for segmenting spleen and liver accurate, there were a limited number of subjects along with ground truth were available. Therefore, we decided to use all the data to train the model for cross validation and did not use a separate 10% for the test set.

## Conclusion

In conclusion, the study has demonstrated the successful development and evaluation of deep learning techniques for 3-slice CT image segmentation, enabling detailed analysis of numerous tissues and organs related to body composition. The four models evaluated showed relatively good results during cross validation and testing, which can reduce analysis time and provide objective results. These findings highlight the potential for automated segmentation results to be used in detailed studies on the relationship between body composition and present and future health data collected in studies using the described 3-slice CT protocol. The results of this study have significant implications for the field of body composition analysis, paving the way for further research and advancements in this area.

### Supplementary Information

**Additional file 1**. Supplementary Materials.

## Data Availability

SCAPIS data will be available to researchers (principal investigator currently needs to be based in Sweden) via the data sharing platform, after ethical approval and a project application and approval. IGT data can be made available for research collaborations after reasonable request to study co-principal investigator G.B. Reference segmentations created at Antaros Medical cannot be shared. Reference segmentation created at Uppsala University can be shared upon request, after project approval from SCAPIS platform.
