# Supplementary material for: Automatic segmentation of large-scale CT image datasets for detailed body composition analysis
Source: BMC Bioinformatics. 2023 Sep 18;24:346. doi: 10.1186/s12859-023-05462-2 (PMC10506248; doi:10.1186/s12859-023-05462-2)
Supplement: Supplementary file 1 — Additional file 1. Supplementary Materials. [file 12859_2023_5462_MOESM1_ESM.docx]

**Supplementary Materials**


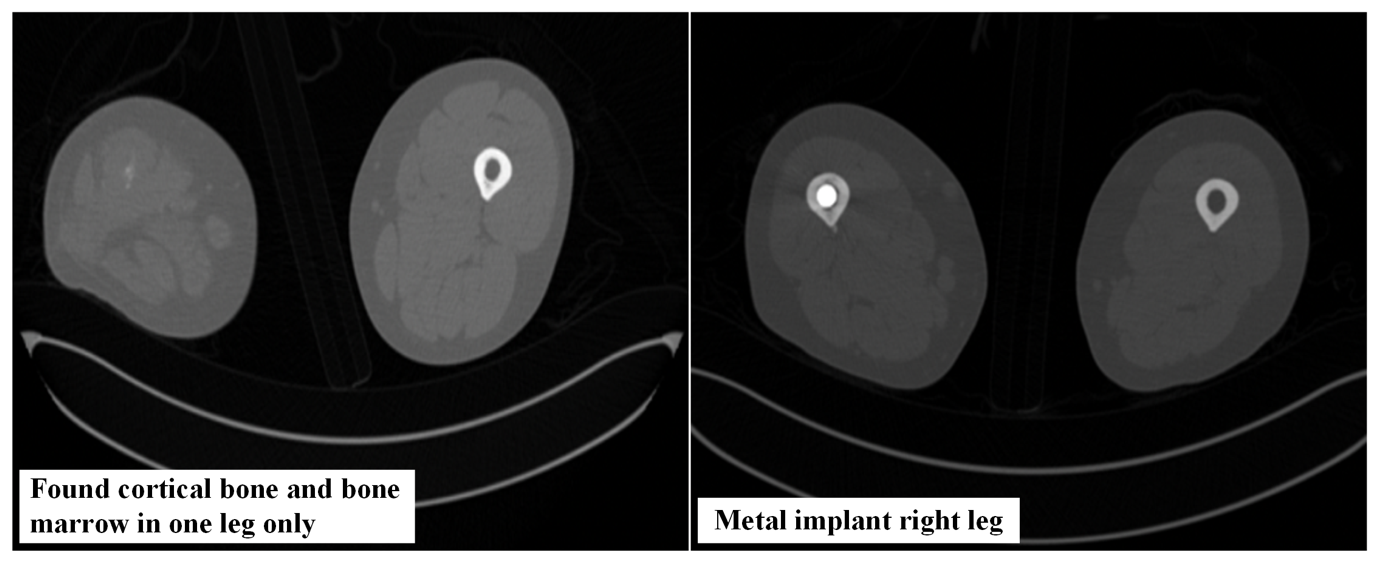


**Additional file 1: Fig. S1** Visualization of anomaly and metal artifact in the thigh CT images.


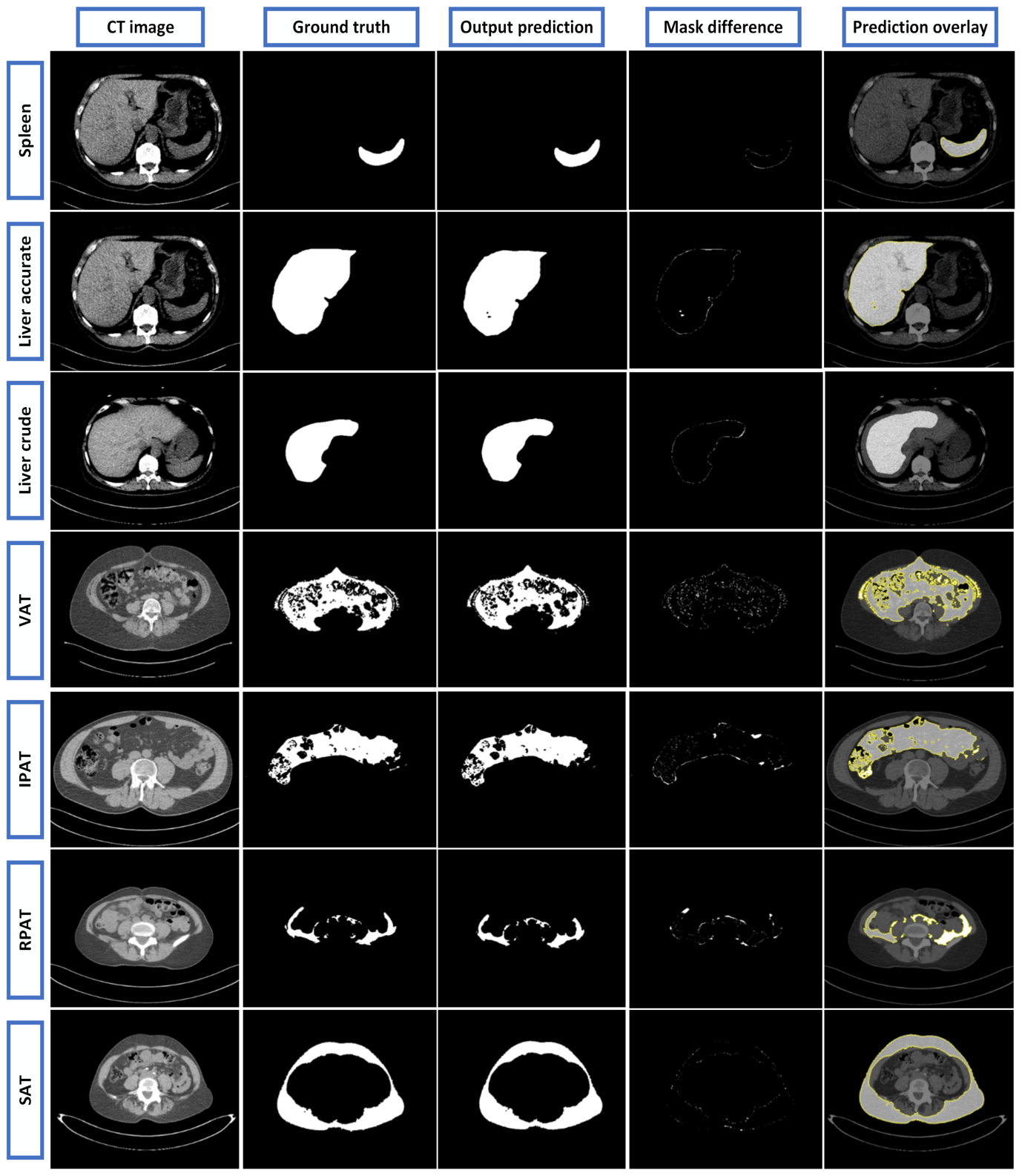


**Additional file 1: Fig. S2(a)** Illustration of ResUNET model predictions and comparison to reference segmentations for randomly selected CT image examples, from top to bottom (Spleen to SAT) images: from left to right, CT image, ground truth, models predicted output, mask difference between ground truth and prediction, predicted mask overlayed on the original CT image, highlighted segmented region (contour) with mark boundaries.


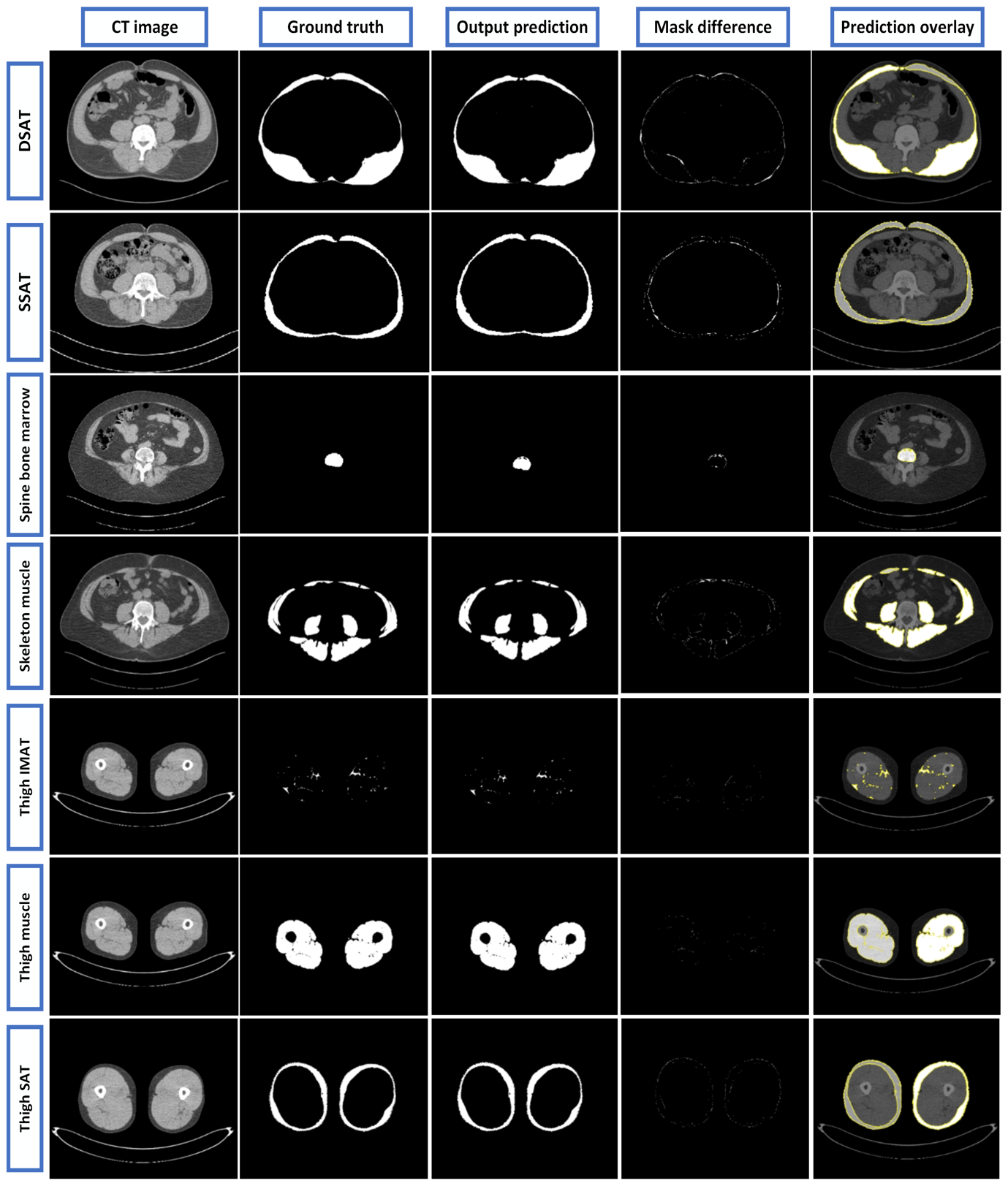


**Additional file 1: Fig. S2(b)** Illustration of ResUNET model predictions and comparison to reference segmentations for randomly selected CT image examples, from top to bottom (DSAT to Thigh SAT) images: from left to right, CT image, ground truth, models output prediction, mask difference between ground truth and prediction, predicted mask overlayed on the original CT image, highlighted segmented region (contour) with mark boundaries.


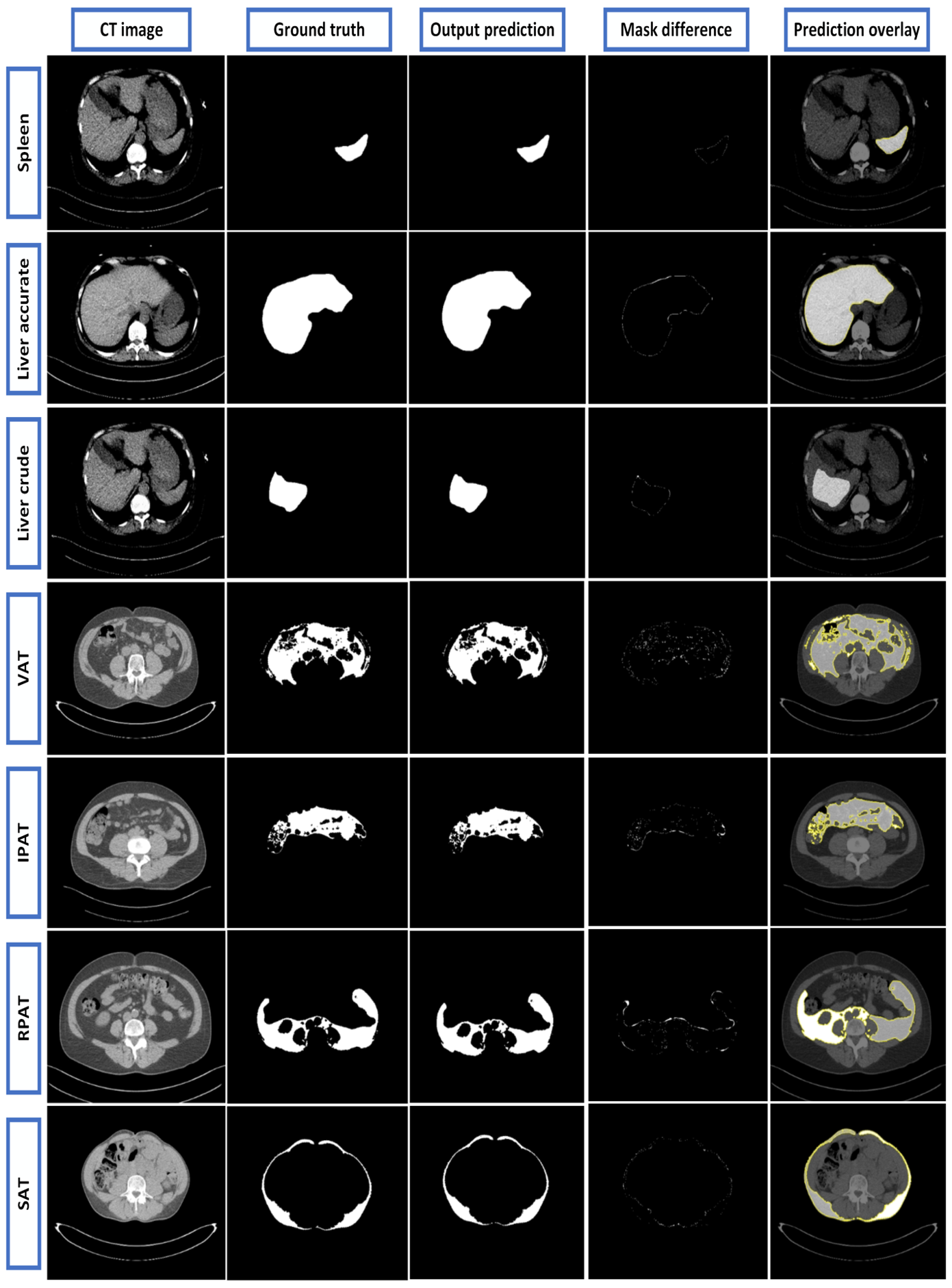


**Additional file 1: Fig. S3(a)** Illustration of Ghost-UNET model predictions and comparison to reference segmentations for randomly selected CT image examples, from top to bottom (Spleen to SAT) images: from left to right, CT image, ground truth, models predicted output, mask difference between ground truth and prediction, predicted mask overlayed on the original CT image, highlighted segmented region (contour) with mark boundaries.


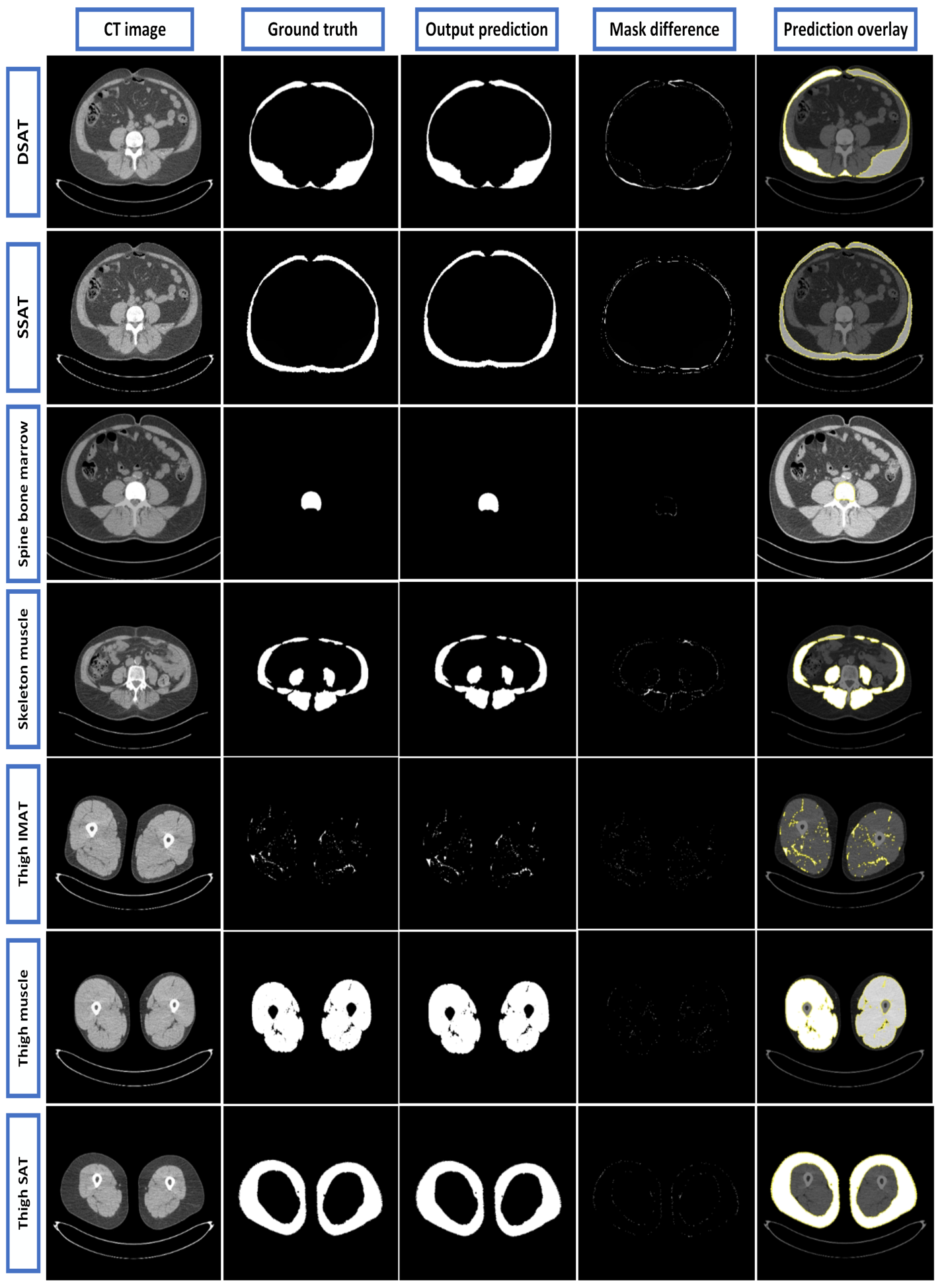


**Additional file 1: Fig. S3(b)** Illustration of Ghost-UNET model predictions and comparison to reference segmentations for randomly selected CT image examples, from top to bottom (DSAT to Thigh SAT) images: from left to right, CT image, ground truth, models output prediction, mask difference between ground truth and prediction, predicted mask overlayed on the original CT image, highlighted segmented region (contour) with mark boundaries.


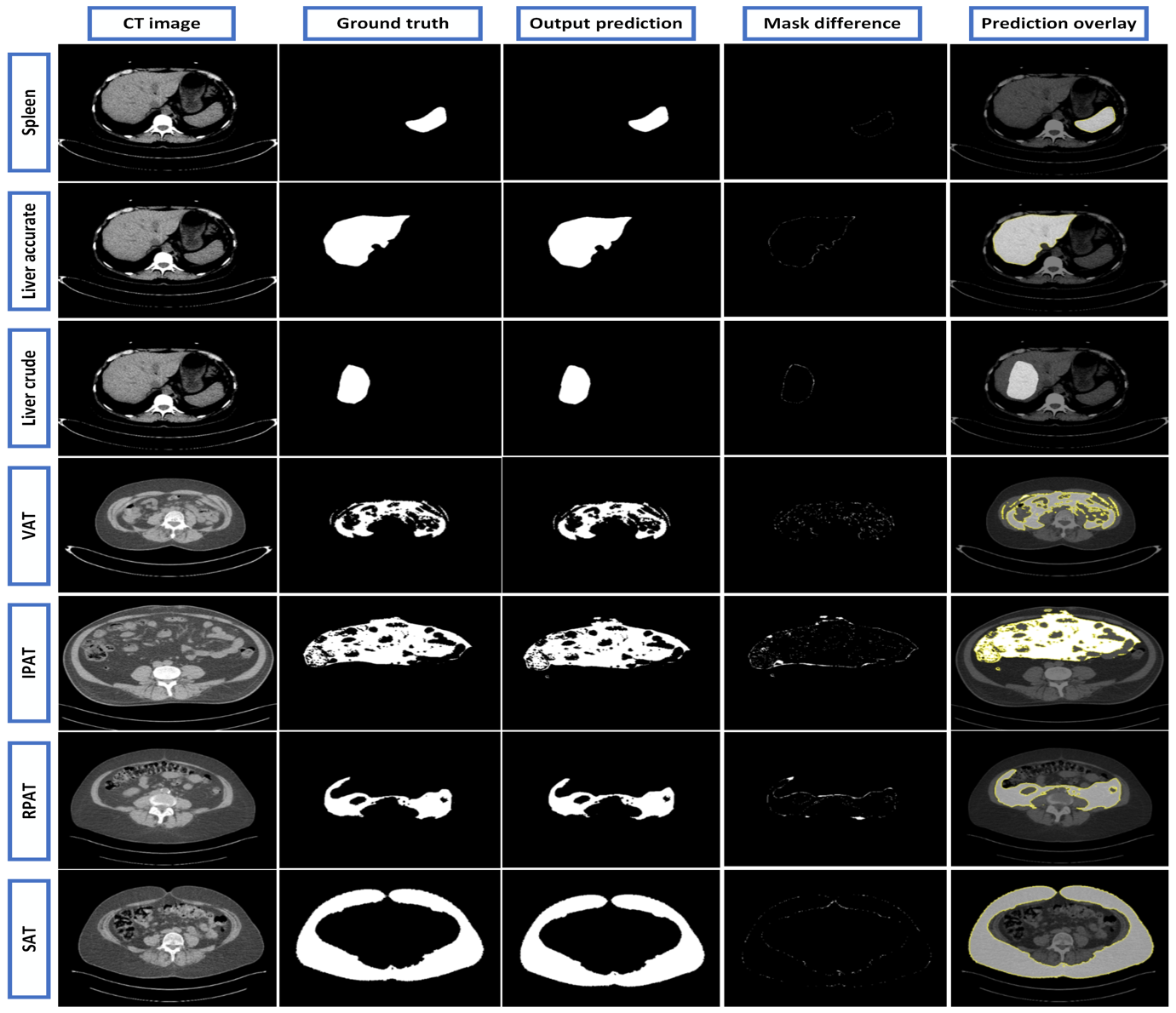


**Additional file 1: Fig. S4(a)** Illustration of Ghost-UNET++ model predictions and comparison to reference segmentations for randomly selected CT image examples, from top to bottom (Spleen to SAT) images: from left to right, CT image, ground truth, models predicted output, mask difference between ground truth and prediction, predicted mask overlayed on the original CT image, highlighted segmented region (contour) with mark boundaries.


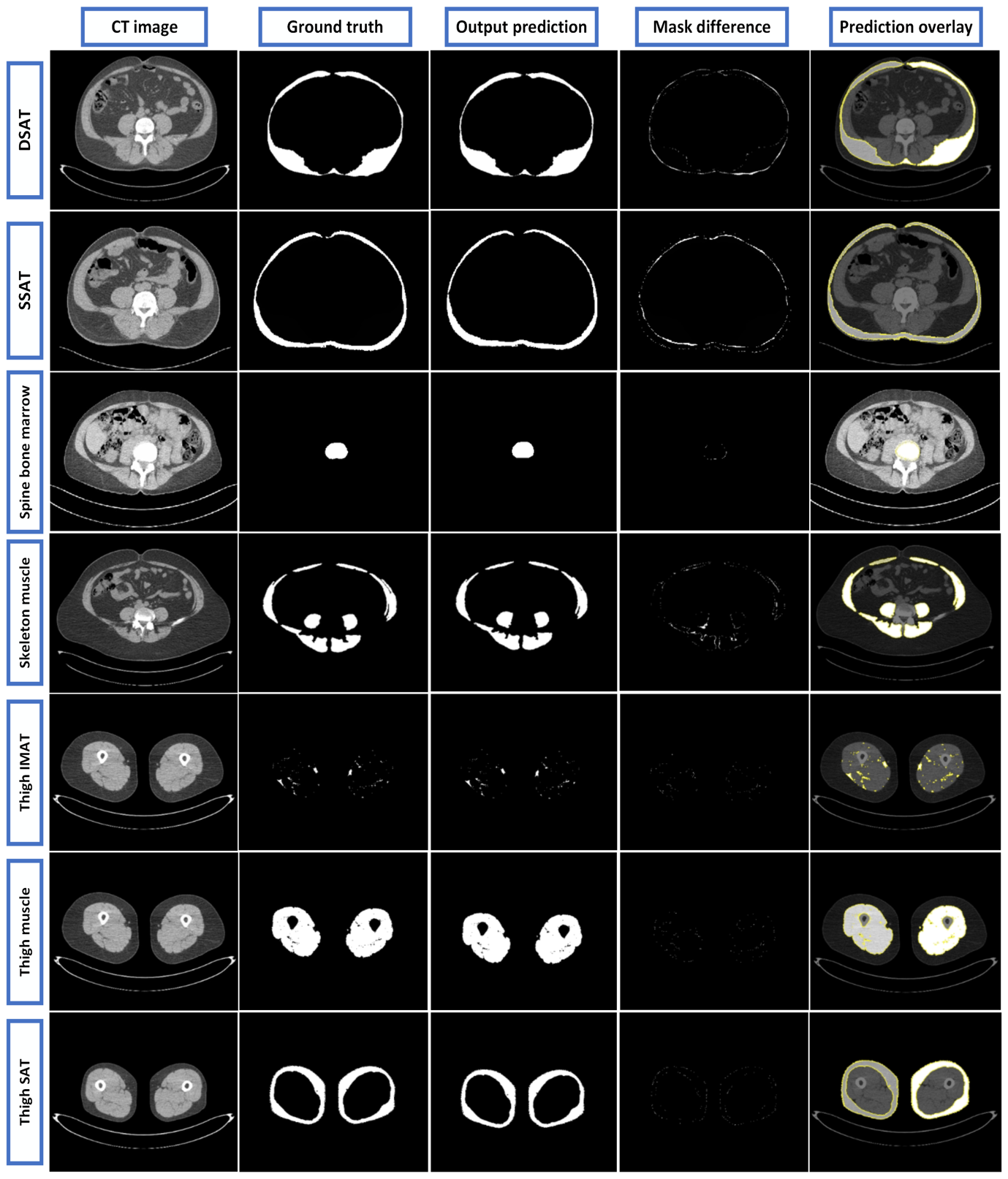


**Additional file 1: Fig. S4(b)** Illustration of Ghost-UNET++ model predictions and comparison to reference segmentations for randomly selected CT image examples, from top to bottom (DSAT to Thigh SAT) images: from left to right, CT image, ground truth, models output prediction, mask difference between ground truth and prediction, predicted mask overlayed on the original CT image, highlighted segmented region (contour) with mark boundaries.
